# Dexmedetomidine as a Ropivacaine Adjuvant in a Thoracic Paravertebral Block Combined With an Erector Spinae Plane Block for Improving Early Quality of Recovery After Transapical Transcatheter Aortic Valve Implantation

**DOI:** 10.1002/kjm2.70058

**Published:** 2025-06-06

**Authors:** Gui‐Ling Dong, Rong‐En Qiu, Chen‐Zhan Xu

**Affiliations:** ^1^ Department of Anesthesiology The Quzhou Affiliated Hospital of Wenzhou Medical University, Quzhou People's Hospital Quzhou China

**Keywords:** dexmedetomidine, erector spinae plane block, quality of recovery, thoracic paravertebral block, transcatheter aortic valve implantation

## Abstract

The study aimed to investigate the effectiveness of dexmedetomidine (Dex) as an adjuvant for ropivacaine in a thoracic paravertebral block (TPVB) combined with an erector spinae plane block (ESPB) for improving early quality of recovery and postoperative pain after transapical transcatheter aortic valve implantation (TAVI). A total of 89 patients who were scheduled to undergo transapical TAVI under general anesthesia were allocated into Group RS and Group RD by using a computer‐generated random‐number list. Group RS (*n* = 45) received 35 mL of 0.5% ropivacaine with 5 mL of a normal saline mixture in TPVB combined with ESPB and Group RD (*n* = 44) received 35 mL of 0.5% ropivacaine with 1 μg/kg Dex in 5 mL of normal saline. The RD group exhibited significantly higher global QoR‐15 scores with lower visual analog scale (VAS) scores at 12 and 24 h postoperatively than the RS group. The RD group needed fewer press times of PCIA than the RS group. The postoperative sufentanil consumption was significantly less in the RD group than that in the RS group. A longer time to first use of flurbiprofen with less remedial doses of flurbiprofen consumption within 48 h postoperatively was required for the RD group than that for the RS group. The two groups did not differ in the incidence of side effects. The findings of the study suggest that adding Dex to ropivacaine in TPVB combined with ESPB is effective in improving early quality of recovery and alleviating postoperative pain for patients undergoing transapical TAVI under general anesthesia.

## 1 Introduction

Transcatheter aortic valve implantation (TAVI) has become the preferred treatment option for patients with symptomatic severe aortic stenosis (AS) at high surgical risks over two decades [1, 2]. As early valve replacement may prevent irreversible cardiac remodeling, TAVI has been indicated in the eligible population expanded to bicuspid aortic valve with feasible anatomy, asymptomatic severe, and moderate AS among all risk subsets [3, 4]. In lower‐risk patients, TAVI was associated with lower risks of bleeding, disabling stroke, atrial fibrillation, and kidney injury, but higher risks of pacemaker implantation and moderate–severe aortic regurgitation compared to surgical aortic valve implantation [5, 6]. Although transfemoral access has been recognized as the first‐line access for conducting TAVI, a diverse patient profile with different risk factors, such as peripheral artery disease, limited the feasibility of a transfemoral access [7]. Transapical TAVI has gained increasing application in patients with AS where transfemoral access is unfavorable or not feasible, showing very promising early and mid‐term outcomes without early/late aortic syndrome [8, 9]. In addition to minimal incision, transapical TAVI exhibits several advantages including shorter procedural distances, operating in the direction of blood flow, and lower incidences of stroke and organ embolisms. However, for many anesthesiologists and thoracic surgeons, early quality of recovery (QoR) with reduced postoperative pain remains a significant challenge [10].

Thoracic paravertebral block (TPVB) has been adopted as the standard analgesia for video‐assisted thoracoscopic surgery (VATS) by accommodating local anesthetic that can spread into all surrounding spaces including cephalad, caudal, intercostal (including the dorsal intercostal compartments), interpleural, epidural, and prevertebral spaces [11, 12]. As for transapical TAVR procedures, TPVB was demonstrated with an opioid‐sparing effect, but it did not significantly reduce the rate of postoperative delirium compared to patient‐controlled intravenous analgesia (PCIA) of opioids [13]. Although TPVB exhibits similar analgesic efficacy like thoracic epidural blockade, single‐injection TPVB showed a failure rate of 13%, and it yielded an unpredictable block [14]. TPVB possibly led to hemodynamic fluctuation, as the incidence of hypotension was 4% [15]. In addition to TPVB, erector spinae plane block (ESPB) has also been recommended for postoperative analgesia after VATS [16] by having the needle endpoints progressively further away from the paravertebral space, intercostal space, and epidural space compartments from an anatomical perspective [17]. However, ESPB alone might cause insufficient analgesia. A study performed on healthy volunteers showed that when ESPB provided a widespread block in the posterior wall of the thorax, only the dorsal nerves were blocked [18]. A cadaveric study about ESPB reported that there was no spread of dye anteriorly to the paravertebral space after ESPB [19]. In alignment with the mechanism of action of multimodal analgesia, the combination of TPVB and ESPB may not only provide a synergistic blockade effect but also reduce the dose of local anesthetic in TPVB, thus reducing the incidence of hypotension. Furthermore, the combination did not make the block more complicated or traumatic, as TPVB and ESPB could be performed with only one puncture in the sagittal section of the ultrasound. Previous literature demonstrated TPVB combined with ESPB provided superior primary and secondary outcomes, such as improved QoR and reduced postoperative pain after minimally invasive thoracic surgery, as compared to TPVB alone [20, 21]. Local anesthetic adjuvants, such as dexmedetomidine (Dex), have been found to prolong the effective duration and enhance the quality of a peripheral nerve block [22]. The study aimed to explore the effect of adding Dex to ropivacaine in TPVB and ESPB combination on early QoR and postoperative pain after transapical TAVR. The researchers hypothesized that ropivacaine with Dex in TPVB combined with ESPB could effectively facilitate early QoR, reduce postoperative pain, decline postoperative sufentanil, and postoperative remedial analgesic drug consumption without adding side effects in patients undergoing transapical TAVR.

## 2 Methods

### 2.1 Participant Selection and Randomization

The study recruited patients who were scheduled to undergo elective transapical TAVI under general anesthesia, with a body mass index (BMI) of 18–30 kg/m^2^, and aged ≥ 18 years. Participant recruitment started in February 2022 and ended in February 2025. The exclusion criteria were refusal to provide written informed consent, severe abnormalities in coagulation function, recent acute pain or chronic pain, the long‐term use of steroids, and allergy to any of the drugs used in the study. The elimination criteria were conversion to open thoracotomy during the perioperative period. Ethical approval for this study was obtained from the Ethics Committee of our hospital. Each participant provided written informed consent. The study protocols were performed in strict accordance with the principles of the Declaration of Helsinki. All patients were randomly allocated into Group RS and Group RD by an independent researcher using a computer‐generated random‐number list with a 1:1 allocation ratio. This independent researcher did not participate in patient recruitment, anesthesia administration, and outcome evaluation. Group RS received 35 mL of 0.5% ropivacaine with 5 mL of a normal saline mixture. Group RD received 35 mL of 0.5% ropivacaine with 1 μg/kg Dex in 5 mL of normal saline. Preoperative interview researchers who were blinded to the trial grouping completed the cold‐pressor task (CPT) and the QoR‐15 questionnaire for patients before surgery. The same team of thoracic physicians who were blinded to the trial grouping performed all of the surgeries. Outcome assessors, surgeons, and nursing staff will remain blinded until the completion of the study analysis. On the day of surgery, the study medication was prepared in a randomized order hidden in an envelope by an anesthesia nurse not involved in the study. The envelope was not opened until the patients had entered the operating room. The attending anesthesiologist was not blinded to the trial grouping due to the nature of the study.

### 2.2 Standard General Anesthesia

After arrival in the operating room, each patient was given standard monitoring including non‐invasive blood pressure (NIBP), heart rate, electrocardiography (ECG), pulse oxygen saturation (SpO_2_), and the bispectral index (BIS). General anesthesia was induced with etomidate 0.2–0.3 mg/kg, sufentanil 0.1–0.3 μg/kg, and rocuronium 0.6–1.0 mg/kg. After intubation, mechanical ventilation was set with no more than 80% oxygen at a tidal volume of 6–8 mL/kg, an end‐tidal carbon dioxide (CO_2_) tension at 35–40 mmHg, and a positive‐end expiratory pressure (PEEP) at 6–8 cm H_2_O. General anesthesia was maintained with intravenous infusion of remifentanil at 0.05–0.2 μg/kg/min and propofol 4–12 mg/kg/h to maintain a BIS value of 40–60. Sufentanil and rocuronium were used as required.

### 2.3 Ultrasound‐Guided TPVB Combined With ESPB

After general anesthesia induction, the patient lying in a standard lateral position with the affected side up received ultrasound‐guided TPVB combined with ESPB. Following skin disinfection, the ultrasound probe was placed at the T5–6 spinous process space of the patient on the affected side. The median sagittal scanning displayed the T5–6 transverse process and pleura, with the paravertebral space presented as a wedge‐shaped hypoechoic region. A 22G sterile puncture needle (0.9 mm × 80 mm) was inserted between the transverse costal process fascia and pleura in the paravertebral space of the thoracic spine. Injection of 20 mL of preconfigured local anesthetic was performed after confirmation of no blood or cerebrospinal fluid observed upon withdrawing the needle. Afterwards, the ultrasonic probe was moved longitudinally 3 cm from the midline of the T5 level of the spine. The puncture needle was penetrated into the trapezius, rhomboid, and erector spinae muscles and stopped between the deep surface of the erector spinae muscle and the T5 transverse processes. After confirmation of no blood or cerebrospinal fluid observed upon withdrawing the needle, injection of 20 mL of preconfigured local anesthetic was performed. The prescription for Group RS was 35 mL of 0.5% ropivacaine with 5 mL of a normal saline mixture, and for Group RD, it was 35 mL of 0.5% ropivacaine with 1 μg/kg Dex in 5 mL of normal saline. Postoperatively, the patient was connected to a PCIA pump which was programmed to intravenously administer 100 μg of sufentanil and 25 mg of dolasetron mesylate diluted to 100 mL with 0.9% normal saline. The PCIA pump was set with a bolus amount of 2 mL, a background dose of 1 mL/h, and a locking time of 15 min. The PCIA devices were removed 48 h after the surgery. The pain degree of patients was assessed using the visual analog scale (VAS), and 50 mg of flurbiprofen was used for remedial analgesia when the VAS score was ≥ 4.

### 2.4 Preoperative and Intraoperative Data Collection

Patient's age, sex, BMI, the presence of hypertension, diabetes mellitus and coronary artery disease, the type of aortic valve disease, aortic valve area, peak aortic flow velocity, New York Heart Association (NYHA) classification, European system for cardiac operative risk evaluation, left ventricular ejection fraction (LVEF), pain threshold and tolerance, surgical duration, sufentanil and remifentanil consumption during surgery were collected. Patient's pain threshold and tolerance were evaluated by the CPT which is a method by placing a hand in a container of cold water to permit slowly increasing pain and is terminated by voluntary withdrawal of the limb [23]. The time (in second) between the start of the immersion and the reporting of the pain they first experienced pain was considered as the pain threshold. The time (in second) between the reporting of first pain and the removal of their hands in the water “until it is too painful.”

### 2.5 Outcome Measures

The primary outcome was the score of the QoR‐15 questionnaire at 12, 24, 48, and 72 h after surgery. The QoR‐15 questionnaire is a validated patient‐centered tool that measures the quality of postoperative recovery, which is comprised of physical comfort (five items), physical independence (two items), psychological support (two items), emotional state (four items), and pain (two items) [24]. A higher global QoR‐15 score (ranging from 0 to 150) reflects better quality of postoperative recovery. The secondary outcomes were the score of VAS at 4, 8, 12, 24, 48, and 72 h after surgery, the total times of analgesic demand on PCIA, the time of first request for flurbiprofen, postoperative sufentanil consumption, the remedial dose of flurbiprofen consumption within 48 h postoperatively, and the incidence of side effects including hypotension, bradycardia, nausea and vomiting, and dizziness. The patients were also taught how to complete the global QoR‐15 questionnaire and the VAS before surgery.

### 2.6 Sample Size Calculation

Sample size calculation was computed with prior power analysis using the G*power software (version 3.1.9.2) and based on the QoR‐15 score. According to a prior publication [25], where an 8‐point difference in the QoR‐15 score is the least difference indicating a clinical improvement or deterioration between two dependent groups, 37 for each group (74 in total) will be required to achieve a power of 90% to detect the difference at a two‐sided *α* level of 0.05. Considering the missing data to be 20%, 45 participants in each group were finally included.

### 2.7 Statistical Analysis

According to the normality of variable distribution confirmed by the Shapiro–Wilk test, the observed results were described as either mean ± standard deviation (SD) or median (quartile 1 [Q1], quartile 3 [Q3]) and analyzed using unpaired *t* test or Mann–Whitney *U* test between two independent groups. Moreover, categorical variables (*n*/%) were analyzed by the chi‐square test. All statistical tests used a two‐tailed *p* < 0.05 as statistically significant in GraphPad Prism, version 6.0 (GraphPad, San Diego, CA, USA).

## 3 Results

### 3.1 Demographic, Clinical, and Intraoperative Characteristics of Participants

**FIGURE 1:**
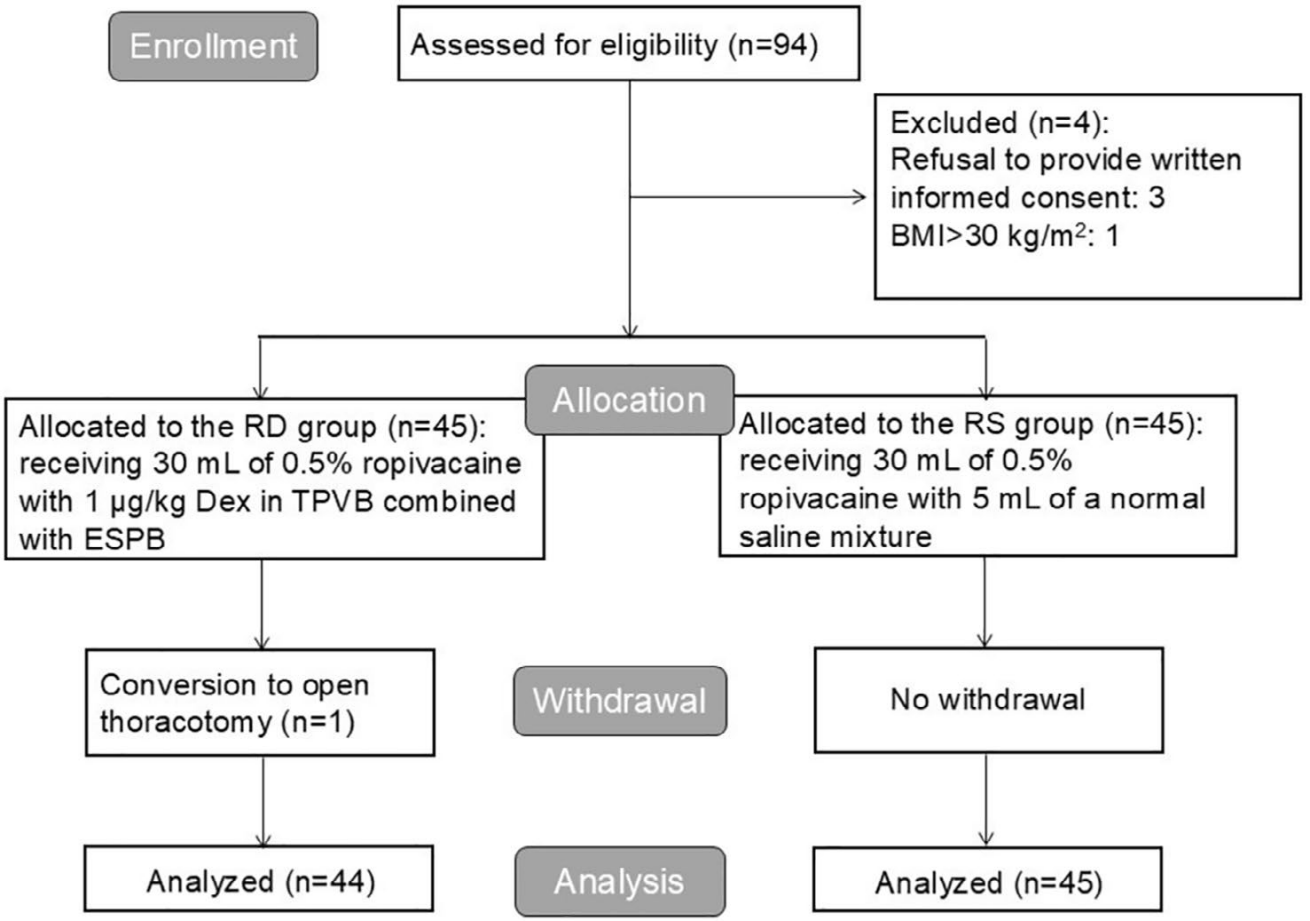
Flowchart of participant recruitment.

A total of 94 patients who scheduled to undergo elective transapical TAVI under general anesthesia were initially selected for this study; 3 were excluded due to their refusal to provide written informed consent, and 1 was excluded due to BMI > 30 kg/m^2^. During the perioperative period, a case of conversion to open thoracotomy occurred in the RD group due to refractory hypotension followed by central venous pressure (CVP) elevation to 33 mmHg 9 min after valve implantation, and this patient was excluded from the final data analysis. Finally, the RD group included 44 patients, and the RS group included 45 patients (Figure 1). Demographic, clinical, and intraoperative characteristics of participants are presented in Table 1. The RD group and RS group exhibited no significant difference regarding demographic and clinical characteristics, surgical duration, and intraoperative sufentanil and remifentanil consumption of participants (*p* > 0.05).

**TABLE 1:** Demographic, clinical, and intraoperative data of participants.

Characteristic	RD (*n* = 44)	RS (*n* = 45)	*p*
Age (year, mean ± SD)	72.6 ± 3.7	73.5 ± 3.9	0.291
Female (*n*/%)	17 (40.9%)	20 (44.4%)	0.831
BMI (kg/m^2^, mean ± SD)	24.4 ± 3.7	24.7 ± 3.1	0.619
Hypertension (*n*/%)	33 (75.0%)	36 (80.0%)	0.620
Diabetes mellitus (*n*/%)	12 (27.3%)	10 (22.2%)	0.630
Coronary artery disease	15 (34.1%)	14 (31.1%)	0.823
Aortic valve disease (*n*/%)			0.666
Aortic stenosis	26 (59.1%)	29 (64.4%)	
Aortic regurgitation	18 (40.9%)	16 (35.6%)	
Aortic valve area (cm^2^, mean ± SD)	0.78 ± 0.10	0.79 ± 0.10	0.741
Peak aortic flow velocity (m/s, mean ± SD)	4.21 ± 0.40	4.15 ± 0.49	0.487
NYHA classification III or IV(*n*/%)	12 (27.3%)	14 (31.1%)	0.816
Log‐EuroSCORE II (%) (*n*/%)			0.893
High risk (> 10%)	2 (4.5%)	2 (4.4%)	
Intermediate risk (5%–10%)	8 (18.2%)	10 (22.2%)	
Low risk (< 5%)	34 (77.3%)	33 (73.3%)	
LVEF (*n*/%)			0.680
> 50%	21 (47.7%)	23 (51.1%)	
30%–50%	19 (43.2%)	20 (44.5%)	
< 30%	4 (9.1%)	2 (4.4%)	
Pain threshold (s, mean ± SD)	38.6 ± 11.3	36.4 ± 11.5	0.376
Pain tolerance (s, mean ± SD)	78.1 ± 17.8	75.1 ± 17.2	0.423
Surgical duration [min, median (Q1, Q3)]	89 (75.8, 115.5)	88 (76, 106)	0.629
Sufentanil consumption (μg)	46.8 ± 3.9	47.9 ± 4.6	0.185
Remifentanil consumption (μg)	668.7 ± 55.0	685.8 ± 60.5	0.159

*Note*: Data summarized as mean ± SD are analyzed by unpaired *t* test. Data summarized as median (Q1, Q3) are analyzed by Mann–Whitney *U* test. Data shown as numbers (percentage) are analyzed by the chi‐square test.

Abbreviations: EuroSCORE, European system for cardiac operative risk evaluation; LVEF, left ventricular ejection fraction; NYHA, New York Heart Association.

### 3.2 Primary Outcomes

The global QoR‐15 scores preoperatively, at 12, 24, 48, and 72 h postoperatively of patients in the RD group and the RS group are summarized in Table 2. Two groups did not differ in preoperative global QoR‐15 scores. The mean value of global QoR‐15 scores at 12 h postoperatively in the RD group was 106.3, and that in the RS group was 95.5, which showed significant differences (*p* < 0.001). The mean value of global QoR‐15 scores at 24 h postoperatively in the RD group was 116.6, and that in the RS group was 107.0, which showed significant differences (*p* < 0.001). With regard to patients' global QoR‐15 scores at 48 and 72 h postoperatively, the two groups did not differ (*p* > 0.05).

**TABLE 2:** The global QoR‐15 scores preoperatively, at 12, 24, 48, and 72 h postoperatively in the RD group and the RS group.

Global QoR‐15 score	RD (*n* = 44)	RS (*n* = 45)	Mean difference (95% CI)	*p*
Preoperative	136.0 ± 3.9	135.2 ± 3.9	0.76 (−0.8 to 2.4)	0.363
Postoperative 12 h	106.3 ± 5.5	95.5 ± 5.0	10.8 (8.6–13.1)	< 0.001
Postoperative 24 h	116.6 ± 8.4	107.0 ± 8.5	9.6 (6.0–13.1)	< 0.001
Postoperative 48 h	119.9 ± 8.1	116.8 ± 9.6	3.2 (0.6–6.9)	0.100
Postoperative 72 h	124.7 ± 9.7	121.6 ± 10.1	3.1 (−1.1 to 7.3)	0.143

*Note*: Data summarized as mean ± SD are analyzed by unpaired *t* test.

Abbreviation: QoR‐15, 15‐item quality of recovery questionnaire.

### 3.3 Secondary Outcomes

The VAS scores at 4, 8, 12, 24, 48, and 72 h postoperatively of patients in the RD group and the RS group are presented in Table 3. The VAS scores at 12 and 24 h postoperatively were notably lower in the RD group than the RS group (*p* = 0.002; *p* = 0.028). As for other time points postoperatively (4, 8, 48, and 72 h), the VAS scores were similar between the RD group and the RS group (*p* > 0.05).

**TABLE 3:** The VAS scores at 4, 8, 12, 24, 48, and 72 h postoperatively in the RD group and the RS group.

VAS score	RD (*n* = 44)	RS (*n* = 45)	*p*
Postoperative 4 h	2 (1, 3)	2 (1, 4)	0.437
Postoperative 8 h	2 (1, 4)	2 (1, 4)	0.674
Postoperative 12 h	2 (2, 4)	3 (2, 4)	0.002
Postoperative 24 h	4 (3, 5)	5 (3, 6)	0.028
Postoperative 48 h	3.5 (2, 4)	3 (2, 4.5)	0.545
Postoperative 72 h	2 (1, 4)	3 (2, 4)	0.182

*Note*: Data summarized as median (Q1, Q3) are analyzed by Mann–Whitney *U* test.

Abbreviation: VAS, visual analog scale.

**FIGURE 2:**
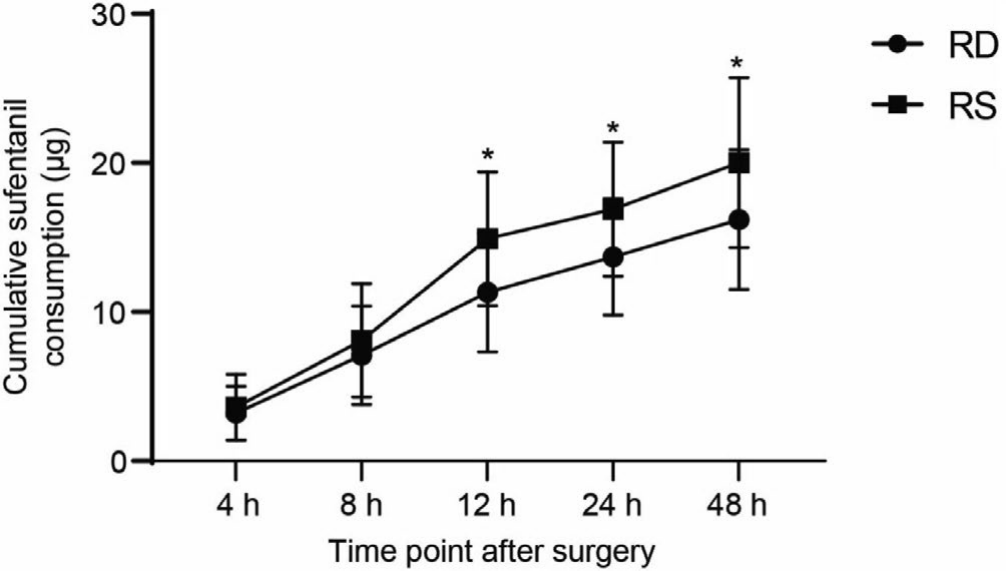
Cumulative sufentanil consumption within 48 h postoperatively between the RD group and RS group. *p < 0.05 by unpaired *t* test.

As listed in Table 4, the RD group needed fewer press times of PCIA than the RS group (*p* = 0.010). The cumulative sufentanil consumption within 48 h postoperatively was significantly less in the RD group than that in the RS group (*p* = 0.001). More specifically, the cumulative sufentanil consumption at 12, 24, and 48 h postoperatively was significantly reduced in the RD group compared with the RS group (*p* < 0.05, Figure 2). A longer time to first use of flurbiprofen with less remedial dose of flurbiprofen consumption within 48 h postoperatively was required for the RD group than that for the RS group (*p* < 0.001; *p* = 0.009). The RD group had 16 cases of patients experiencing side effects, including hypotension (*n* = 4), bradycardia (*n* = 5), nausea and vomiting (*n* = 3), or dizziness (*n* = 4). The RS group had 17 cases of patients experiencing side effects, including hypotension (*n* = 2), bradycardia (*n* = 2), nausea and vomiting (*n* = 7), or dizziness (*n* = 6). The two groups did not differ in the incidence of side effects. Additionally, none of the patients experienced adverse events related to TPVB or ESPB.

**TABLE 4:** Analysis of secondary outcomes between the RD group and the RS group.

	RD (*n* = 44)	RS (*n* = 45)	*p*
Total press time of PCIA	7.5 (5.25, 11)	10 (7, 13)	0.010
Postoperative sufentanil consumption (μg)	16.2 ± 4.7	20.0 ± 5.7	0.002
Time to first flurbiprofen (h)	19.2 ± 4.1	13.8 ± 2.8	< 0.001
Remedial dose of flurbiprofen (mg)	46.3 ± 24.4	59.7 ± 27.8	0.009
Side effects [case number (%)]	16 (36.4%)	17 (37.8%)	0.890
Hypotension	4	2	
Bradycardia	5	2	
Nausea and vomiting	3	7	
Dizziness	4	6	

*Note*: Data summarized as mean ± SD are analyzed by unpaired *t* test. Data summarized as median (Q1, Q3) are analyzed by Mann–Whitney *U* test. Data shown as numbers (percentage) are analyzed by the chi‐square test.

## 4 Discussion

The main findings of our study were that adding Dex to ropivacaine in TPVB and ESPB combination could effectively improve the QoR and reduce postoperative pain at 12 and 24 h after surgery, showing opioid‐sparing postoperative analgesia but without adding side effects for patients undergoing transapical TAVR.

Combined regional block techniques have been shown to be effective and safe in improving postoperative analgesia, which become crucial components of multimodal analgesia for cardiothoracic surgeries, such as the combined serratus anterior plane block and transverse thoracic muscle plane block for coronary artery bypass surgery [26] and the combined deep and superficial serratus anterior plane block for VATS [27]. When TPVB and ESPB are combined, the needle path to the thoracic paravertebral space could enhance the diffusion of local anesthetic reserved in the erector spinae plane, which would potentiate the certainty of analgesia and reduce the side effect of nerve block. With regard to procedure, the researchers should be proficient in maintaining consistency and accuracy in drug administration. Therefore, establishment of ongoing training and certification processes is crucial to ensure specialized training and expertise when performing ultrasound‐guided combination of TPVB and ESPB. In our study, no complications such as bleeding or hematoma have been observed, indicating that a combination of TPVB and ESPB can be safely implemented in patients undergoing transapical TAVI. Not consistent with our results, Zhang et al. [28] found that the combined TPVB and ESPB did not exhibit superior analgesic effects but led to a faster sensory loss after nerve block with reduced incidence of postoperative chronic pain compared to ESPB alone in patients undergoing VATS. Adding Dex as an adjunct to local anesthetic in this technique may explain the superior analgesic effects observed in our study.

The peripheral analgesic effect of Dex supports its addition to ropivacaine in a combination of TPVB and ESPB rather than intravenous access [29]. Dex augments regional anesthesia by prolonging sensory blockade and reducing central sensitization by its dual action on presynaptic inhibition of nociceptive neurotransmitter release and postsynaptic hyperpolarization of spinal neurons. Adding Dex as a ropivacaine adjuvant, ESPB was demonstrated with the ability to improve the postoperative QoR and prolong postoperative analgesia for patients undergoing video‐assisted thoracoscopic lobectomy surgery [30]. Moreover, the addition of Dex, 1 μg/kg, to ropivacaine in multilevel TPVB was shown to prolong the duration of postoperative analgesia and improve patient satisfaction in patients undergoing VATS [31]. While Dex is widely recognized for its analgesic and sedative properties, there is an emerging interest in other adjuvants, such as dexamethasone. Yang et al. [32] have evaluated the analgesic efficacy of dexamethasone versus Dex as adjuvants to ropivacaine during TPVB combined with ESPB and reported similar postoperative analgesia after VATS. However, Tang et al. [33] and Gao et al. [34] reported more effective analgesic effects of Dex over dexamethasone during TPVB or ESPB after VATS. It would be clinically beneficial for further investigations comparing the analgesic efficacy of dexamethasone versus Dex as adjuvants to ropivacaine during TPVB combined with ESPB after transapical TAVI.

The QoR‐15 has excellent reliability and clinical feasibility as a metric of QoR during enhanced recovery after surgery (ERAS) protocols, and it is moderately associated with the incidence of postoperative complications up to 30 days after elective surgeries [35]. Adding Dex to ropivacaine in the combined TPVB and ESPB showed beneficial effects on early QoR and opioid‐sparing perioperative management without increasing pain scores, fostering the adoption of ERAS protocols tailored for transapical TAVI. Opioid‐sparing multimodal analgesia involving paravertebral catheter techniques or erector spinae catheter may be an important component of ERAS protocols for cardiothoracic surgeries, which may result in improvements in functional recovery, length of stay, opioid use, complications, and readmissions [36, 37].

We acknowledge several limitations in this study. First, a relatively small sample size in a single center and participants remaining as a specific population not amenable to transfemoral interventions should be noted. Possible multicenter trials with diverse patient populations would highlight the applicability of our findings to a broader clinical setting. In addition, while we explore the effectiveness of SAPB, uncertainty persists regarding the optimal concentration and dosage of local anesthetics and adjuvants in the combined TPVB and ESPB. Further investigations by setting different doses of Dex or different drugs, such as dexamethasone as adjuncts to local anesthetic to achieve early QoR and opioid‐sparing perioperative management for transapical TAVI, are warranted.

In conclusion, our study suggests that adding Dex to ropivacaine in a combination of TPVB and ESPB could effectively improve the QoR and postoperative pain at 12 and 24 h postoperatively and provide opioid‐sparing postoperative analgesia but without adding side effects for patients undergoing transapical TAVI under general anesthesia. Our study coincides well with the unique value for the implementation of multimode analgesia and the adoption of ERAS protocols in this specific patient population.

## Conflicts of Interest

The authors declare no conflicts of interest.

## Data Availability

Data sharing not applicable to this article as no datasets were generated or analyzed during the current study.

## References

[1] J. Ponte Monteiro , S. Brugaletta , X. Freixa , A. Regueiro , D. Rijo , and M. Sabate , “The Roadmap to Transcatheter Aortic Valve Implantation,” Revista Portuguesa de Cardiologia 42, no. 8 (2023): 733–739. 36948456 10.1016/j.repc.2022.09.004

[2] A. Cribier , H. Eltchaninoff , A. Bash , et al., “Percutaneous Transcatheter Implantation of an Aortic Valve Prosthesis for Calcific Aortic Stenosis: First Human Case Description,” Circulation 106, no. 24 (2002): 3006–3008. 12473543 10.1161/01.cir.0000047200.36165.b8

[3] F. J. Beerkens , G. H. L. Tang , A. S. Kini , et al., “Transcatheter Aortic Valve Replacement Beyond Severe Aortic Stenosis: JACC State‐of‐the‐Art Review,” Journal of the American College of Cardiology 85, no. 9 (2025): 944–964. 40044299 10.1016/j.jacc.2024.11.051

[4] S. K. Brar , D. W. Leong , R. R. Razi , et al., “Early Aortic Valve Replacement in Asymptomatic Severe Aortic Stenosis: A Meta‐Analysis of Randomized Controlled Trials,” American Journal of Cardiology 245 (2025): 11–16. 40054514 10.1016/j.amjcard.2025.02.025

[5] T. T. Lerman , N. Greenberg , M. Kheifets , et al., “Transcatheter Aortic Valve Implantation Versus Surgical Aortic Valve Replacement in Patients at Lower Surgical Risk: Meta‐Analysis of Randomized Trials,” Canadian Journal of Cardiology (2025): S0828‐282X(25)00183‐7. 10.1016/j.cjca.2025.02.036 40074146

[6] R. K. Reddy , J. P. Howard , M. J. Mack , et al., “Transcatheter vs Surgical Aortic Valve Replacement in Lower‐Risk Patients: An Updated Meta‐Analysis of Randomized Controlled Trials,” Journal of the American College of Cardiology 85, no. 9 (2025): 926–940. 40044297 10.1016/j.jacc.2024.12.031

[7] M. W. Abdelnour , V. Patel , P. M. Patel , A. M. Kasel , and A. H. Frangieh , “Alternative Access in Transcatheter Aortic Valve Replacement‐An Updated Focused Review,” Frontiers in Cardiovascular Medicine 11 (2024): 1437626. 39175626 10.3389/fcvm.2024.1437626 PMC11338806

[8] D. Useini , B. Beluli , H. Christ , et al., “Transcatheter Aortic Valve Implantation in Patients Who Cannot Undergo Transfemoral Access,” Thoracic and Cardiovascular Surgeon 70, no. 3 (2022): 189–198. 33851408 10.1055/s-0041-1727131

[9] D. Useini , B. Beluli , H. Christ , et al., “Transapical Transcatheter Aortic Valve Implantation in Patients With Aortic Diseases,” European Journal of Cardio‐Thoracic Surgery 59, no. 6 (2021): 1174–1181. 33709139 10.1093/ejcts/ezab050

[10] G. McCalmont , E. Durand , S. Lauck , et al., “Setting a Benchmark for Resource Utilization and Quality of Care in Patients Undergoing Transcatheter Aortic Valve Implantation in Europe‐Rationale and Design of the International BENCHMARK Registry,” Clinical Cardiology 44, no. 10 (2021): 1344–1353. 34499383 10.1002/clc.23711 PMC8495089

[11] K. Slinchenkova , K. Lee , S. Choudhury , D. Sundarapandiyan , and K. Gritsenko , “A Review of the Paravertebral Block: Benefits and Complications,” Current Pain and Headache Reports 27, no. 8 (2023): 203–208. 37294514 10.1007/s11916-023-01118-1

[12] A. E. Ardon , J. Lee , C. D. Franco , K. T. Riutort , and R. A. Greengrass , “Paravertebral Block: Anatomy and Relevant Safety Issues,” Korean Journal of Anesthesiology 73, no. 5 (2020): 394–400. 32172551 10.4097/kja.20065 PMC7533185

[13] E. Strike , B. Arklina , P. Stradins , et al., “Postoperative Pain Management Strategies and Delirium After Transapical Aortic Valve Replacement: A Randomized Controlled Trial,” Journal of Cardiothoracic and Vascular Anesthesia 33, no. 6 (2019): 1668–1672. 30559067 10.1053/j.jvca.2018.11.010

[14] S. Cheema , J. Richardson , and P. McGurgan , “Factors Affecting the Spread of Bupivacaine in the Adult Thoracic Paravertebral Space,” Anaesthesia 58, no. 7 (2003): 684–687. 12886912 10.1046/j.1365-2044.2003.03189_1.x

[15] Z. Naja and P. A. Lonnqvist , “Somatic Paravertebral Nerve Blockade. Incidence of Failed Block and Complications,” Anaesthesia 56, no. 12 (2001): 1184–1188. 11736777 10.1046/j.1365-2044.2001.02084-2.x

[16] S. Feray , J. Lubach , G. P. Joshi , F. Bonnet , M. Van de Velde , and PROSPECT Working Group of the European Society of Regional Anaesthesia and Pain Therapy , “PROSPECT Guidelines for Video‐Assisted Thoracoscopic Surgery: A Systematic Review and Procedure‐Specific Postoperative Pain Management Recommendations,” Anaesthesia 77, no. 3 (2022): 311–325. 34739134 10.1111/anae.15609 PMC9297998

[17] H. Lim , C. Mathew , S. N. Wong , and C. W. Liu , “Anatomical Insights Into Injectate Spread After Thoracic Erector Spinae Plane Block: A Systematic Review,” Journal of Clinical Anesthesia 92 (2023): 111304. 39491273 10.1016/j.jclinane.2023.111304

[18] J. Zhang , Y. He , S. Wang , et al., “The Erector Spinae Plane Block Causes Only Cutaneous Sensory Loss on Ipsilateral Posterior Thorax: A Prospective Observational Volunteer Study,” BMC Anesthesiology 20, no. 1 (2020): 88. 32312233 10.1186/s12871-020-01002-0 PMC7169010

[19] J. Ivanusic , Y. Konishi , and M. J. Barrington , “A Cadaveric Study Investigating the Mechanism of Action of Erector Spinae Blockade,” Regional Anesthesia and Pain Medicine 43, no. 6 (2018): 567–571. 29746445 10.1097/AAP.0000000000000789

[20] A. Moorthy , A. Ni Eochagain , E. Dempsey , et al., “Postoperative Recovery With Continuous Erector Spinae Plane Block or Video‐Assisted Paravertebral Block After Minimally Invasive Thoracic Surgery: A Prospective, Randomised Controlled Trial,” British Journal of Anaesthesia 130, no. 1 (2023): e137–e147. 36109206 10.1016/j.bja.2022.07.051

[21] M. Zengin , A. Alagoz , H. Sazak , G. Ulger , R. Baldemir , and M. Senturk , “Comparison of Efficacy of Erector Spinae Plane Block, Thoracic Paravertebral Block, and Erector Spinae Plane Block and Thoracic Paravertebral Block Combination for Acute Pain After Video‐Assisted Thoracoscopic Surgery: A Randomized Controlled Study,” Minerva Anestesiologica 89, no. 3 (2023): 138–148. 35766959 10.23736/S0375-9393.22.16639-3

[22] Z. Chen , Z. Liu , C. Feng , Y. Jin , and X. Zhao , “Dexmedetomidine as an Adjuvant in Peripheral Nerve Block,” Drug Design, Development and Therapy 17 (2023): 1463–1484. 37220544 10.2147/DDDT.S405294 PMC10200118

[23] C. L. von Baeyer , T. Piira , C. T. Chambers , M. Trapanotto , and L. K. Zeltzer , “Guidelines for the Cold Pressor Task as an Experimental Pain Stimulus for Use With Children,” Journal of Pain 6, no. 4 (2005): 218–227. 15820909 10.1016/j.jpain.2005.01.349

[24] X. Xu , J. An , Y. Zhang , L. Liu , Y. Chen , and R. Gong , “Investigation of the Quality of Recovery of Surgical Patients Based on the Chinese Version of the Quality of Recovery‐15 Survey, a Cross‐Sectional Study,” Journal of Perianesthesia Nursing 37, no. 2 (2022): 199–203. 34916135 10.1016/j.jopan.2021.04.002

[25] P. S. Myles , D. B. Myles , W. Galagher , C. Chew , N. MacDonald , and A. Dennis , “Minimal Clinically Important Difference for Three Quality of Recovery Scales,” Anesthesiology 125, no. 1 (2016): 39–45. 27159009 10.1097/ALN.0000000000001158

[26] O. C. Yasar , S. Batcik , H. Kazdal , L. Kazancioglu , D. Hemsinli , and B. Erdivanli , “Comparison of the Efficacy of Two Different Plane Blocks in Isolated Bypass Surgery: A Prospective Observational Study,” Journal of Cardiothoracic and Vascular Anesthesia 36, no. 12 (2022): 4333–4340. 36100497 10.1053/j.jvca.2022.08.002

[27] M. Zengin , H. Sazak , R. Baldemir , G. Ulger , and A. Alagoz , “The Effect of Erector Spinae Plane Block and Combined Deep and Superficial Serratus Anterior Plane Block on Acute Pain After Video‐Assisted Thoracoscopic Surgery: A Randomized Controlled Study,” Journal of Cardiothoracic and Vascular Anesthesia 36, no. 8 (2022): 2991–2999. 35249833 10.1053/j.jvca.2022.01.048

[28] L. Zhang , Y. Hu , H. Liu , et al., “Analgesic Efficacy of Combined Thoracic Paravertebral Block and Erector Spinae Plane Block for Video‐Assisted Thoracic Surgery: A Prospective Randomized Clinical Trial,” Medical Science Monitor 29 (2023): e940247. 37408302 10.12659/MSM.940247 PMC10334846

[29] Z. Chen , C. Gao , Y. Zhang , et al., “Effects of Ultrasound‐Guided Thoracic Paravertebral Nerve Block Combined With Perineural or IV Dexmedetomidine on Acute and Chronic Pain After Thoracoscopic Resection of Lung Lesions: A Double‐Blind Randomized Trial,” Drug Design, Development and Therapy 18 (2024): 2089–2101. 38882043 10.2147/DDDT.S457334 PMC11177863

[30] Y. Guo , J. Wang , P. Jiang , D. Wang , W. Fan , and X. Yang , “Effect of Erector Spinae Plane Block With Different Doses of Dexmedetomidine as Adjuvant for Ropivacaine on the Postoperative Quality of Recovery After Video‐Assisted Thoracoscopic Lobectomy Surgery: A Randomized Controlled Trial,” BMC Anesthesiology 23 (2023): 264. 37550610 10.1186/s12871-023-02231-9 PMC10405441

[31] J. Xu , X. Yang , X. Hu , X. Chen , J. Zhang , and Y. Wang , “Multilevel Thoracic Paravertebral Block Using Ropivacaine With/Without Dexmedetomidine in Video‐Assisted Thoracoscopic Surgery,” Journal of Cardiothoracic and Vascular Anesthesia 32, no. 1 (2018): 318–324. 29191649 10.1053/j.jvca.2017.06.023

[32] J. Yang , M. Zhao , X. R. Zhang , et al., “Ropivacaine With Dexmedetomidine or Dexamethasone in a Thoracic Paravertebral Nerve Block Combined With an Erector Spinae Plane Block for Thoracoscopic Lobectomy Analgesia: A Randomized Controlled Trial,” Drug Design, Development and Therapy 16 (2022): 1561–1571. 35655534 10.2147/DDDT.S366428 PMC9152436

[33] R. Tang , W. S. Lu , H. L. Zhong , F. Wu , and Y. Q. Liu , “Comparison of Different Adjuvant Analgesia for Paravertebral Block in Video‐Assisted Thoracoscopic Surgery: A Double‐Blind Randomized Controlled Trial,” PLoS One 20, no. 5 (2025): e0322589. 40315237 10.1371/journal.pone.0322589 PMC12047843

[34] Z. Gao , Y. Xiao , Q. Wang , and Y. Li , “Comparison of Dexmedetomidine and Dexamethasone as Adjuvant for Ropivacaine in Ultrasound‐Guided Erector Spinae Plane Block for Video‐Assisted Thoracoscopic Lobectomy Surgery: A Randomized, Double‐Blind, Placebo‐Controlled Trial,” Annals of Translational Medicine 7, no. 22 (2019): 668. 31930069 10.21037/atm.2019.10.74 PMC6944602

[35] M. Campfort , C. Cayla , S. Lasocki , E. Rineau , and M. Leger , “Early Quality of Recovery According to QoR‐15 Score Is Associated With One‐Month Postoperative Complications After Elective Surgery,” Journal of Clinical Anesthesia 78 (2022): 110638. 35033845 10.1016/j.jclinane.2021.110638

[36] M. Sola , C. J. Ramm , L. M. Kolarczyk , et al., “Application of a Multidisciplinary Enhanced Recovery After Surgery Pathway to Improve Patient Outcomes After Transcatheter Aortic Valve Implantation,” American Journal of Cardiology 118, no. 3 (2016): 418–423. 27344271 10.1016/j.amjcard.2016.05.015

[37] A. Moorthy , A. N. Eochagain , E. Dempsey , and D. Buggy , “Ultrasound‐Guided Erector Spinae Plane Catheter Versus Video‐Assisted Paravertebral Catheter Placement in Minimally Invasive Thoracic Surgery: Comparing Continuous Infusion Analgesic Techniques on Early Quality of Recovery, Respiratory Function and Chronic Persistent Surgical Pain: Study Protocol for a Double‐Blinded Randomised Controlled Trial,” Trials 22, no. 1 (2021): 965. 34963493 10.1186/s13063-021-05863-9 PMC8715598

